# Biomarkers of cardio-renal syndrome in uremic myocardiopathy animal model

**DOI:** 10.1590/2175-8239-JBN-3878

**Published:** 2018-05-07

**Authors:** Laura Mattana Dionísio, Mateus Justi Luvizoto, Caroline Gribner, Danielle Carneiro, Viviane Carvalho, Franciele Robes, Marcos Sheidemantel, Fabiane Rego, Lúcia de Noronha, Roberto Pecoits-Filho, Aline Borsato Hauser

**Affiliations:** 1 Departamento de Análise Clínica, Universidade Federal do Paraná, Curitiba, PR, Brasil.; 2 Faculdade de Medicina, Pontifícia Universidade Católica do Paraná, Curitiba, PR, Brasil.

**Keywords:** kidney diseases, cardio-renal syndrome, tissue microarray, cardiac biomarkers, doenças renais, síndrome cardiorrenal, análises imunoistoquímicas, marcadores cardíacos

## Abstract

**Introduction:**

Cardio-renal syndrome subtype 4 (CRS4) is a condition of primary chronic kidney disease that leads to reduction of cardiac function, ventricular hypertrophy, and risk of cardiovascular events. Objective: Our aim was to understand the mechanisms involved on the onset of CRS4.

**Methods:**

We used the nephrectomy 5/6 (CKD) animal model and compared to control (SHAM). Serum biomarkers were analyzed at baseline, 4, and 8 weeks. After euthanasia, histology and immunohistochemistry were performed in the myocardium.

**Results:**

Troponin I (TnI) was increased at 4 weeks (W) and 8W, but nt-proBNP showed no difference. The greater diameter of cardiomyocytes indicated left ventricular hypertrophy and the highest levels of TNF-α were found at 4W declining in 8W while fibrosis was more intense in 8W. Angiotensin expression showed an increase at 8W.

**Conclusions:**

TnI seems to reflect cardiac injury as a consequence of the CKD however nt-proBNP did not change because it reflects stretching. TNF-α characterized an inflammatory peak and fibrosis increased over time in a process connecting heart and kidneys. The angiotensin showed increased activity of the renin-angiotensin axis and corroborates the hypothesis that the inflammatory process and its involvement with CRS4. Therefore, this animal study reinforces the need for renin-angiotensin blockade strategies and the control of CKD to avoid the development of CRS4.

## RESUMO

**Introdução:**

A síndrome cardiorrenal (SCR) tipo 4 é uma afecção da doença renal crônica primária que leva a redução da função cardíaca, hipertrofia ventricular e risco de eventos cardiovasculares. Objetivo: O objetivo do presente estudo foi compreender os mecanismos envolvidos no surgimento da SCR tipo 4.

**Métodos:**

Um modelo animal de nefrectomia 5/6 (DRC) foi comparado a animais de controle (Placebo). Biomarcadores séricos foram analisados no início do estudo e com quatro e oito semanas de estudo. Após eutanásia, foram realizados exames histológicos e de imunoistoquímica no tecido miocárdico.

**Resultados:**

Troponina I (TnI) estava aumentada nas semanas quatro (S4) e oito (S8), mas o NT-proBNP não apresentou diferenças. O diâmetro maior dos cardiomiócitos indicava hipertrofia ventricular esquerda. Os níveis mais elevados de TNF-α foram identificados na S4 com redução na S8, enquanto fibrose foi mais intensa na S8. A expressão de angiotensina mostrou elevação na S8.

**Conclusões:**

TnI parece sugerir lesões cardíacas em consequência da DRC, porém o NT-proBNP não sofreu alterações por refletir alongamento. O TNF-α evidenciou um pico inflamatório e a fibrose aumentou ao longo do tempo devido ao processo de conexão entre rins e coração. A angiotensina mostrou aumento da atividade do eixo renina-angiotensina, corroborando a hipótese do processo inflamatório e seu envolvimento com SCR tipo 4. Portanto, o presente estudo em modelo animal reforça a necessidade de em adotar estratégias com bloqueadores de renina-angiotensina e controle da DRC para evitar o desenvolvimento de SCR tipo 4.

## INTRODUCTION

The cardio-renal syndrome (CRS) includes a variety of acute or chronic conditions in which the primary failing organ can be either the heart or the kidney.^1^ Direct or indirect dysfunctional effects of each organ can initiate and perpetuate the combined disorder through a complex combination of neurohormonal feedback mechanisms. To cover the vast array of interrelated derangements and stress the bidirectional nature of heart-kidney interactions, Ronco *et al*. presented a classification of the CRS with 5 subtypes based on pathophysiology, the time-frame, and the nature of concomitant cardiac and renal dysfunction providing a more concise and logical approach.^2^ The focus of this study was CRS subtype 4 (CRS4), which is characterized by a condition of primary chronic kidney disease (CKD), which leads to reduction of the cardiac function, ventricular hypertrophy, diastolic dysfunction, and increased risk of adverse cardiovascular events. Patients with CKD are at extremely high cardiovascular risk. CKD is divided into 5 stages based on the combination of kidney damage severity and glomerular filtration rate (GFR) reduction. More than 50% of deaths in stage 5 cohorts are attributed to cardiovascular disease,^1^ but the mechanisms underlying the CRS within of the context of CKD are not well understood.

This animal study aimed to establish a uremic myocardiopathy model for assessment of the natural history of CRS. Some authors suggest that most of the recent advances in the understanding of CRS4 have focused on atherosclerosis and arteriosclerosis, and much less effort has been devoted at evaluating the mechanisms of interventions related to myocardial dysfunction. The echocardiographic evaluation plays a pivotal role in establishing the diagnosis of myocardiopathy as well as in stratifying risk and defining the impact of interventions.^2^ Nevertheless, the investigation of biomarkers and improvement of technologies related to CRS4 are needed.

Of the studied biomarkers, NT-proBNP is produced in the ventricles after stimulus from the stretching of cardiac myocytes in response to cardiac wall stress.^3^^,^ 4 Troponin isoform I (TnI), a protein specific of the cardiac muscle, have been used as auxiliary biomarker in the diagnosis of pathologies that involve necrosis and injury of the myocardial cells.^5^ Regarding cardiac tissue analysis, studies show that CKD patients can develop left ventricular hypertrophy (LVH) and myocardial fibrosis independent of traditional factors, with involvement of the renin-angiotensin-aldosterone system. The inflammatory response has been cited as an important factor in CKD. A study using an animal model found higher TNF-α levels in the uremic group and other study found a relationship between cytokines and deleterious effects on the left ventricle, accelerating the development of heart failure and inducing a hypertrophic response in myocytes.^6^^-^ 8 Thus, reactive oxygen species with high oxidizing potential are generated leading to oxidative stress.^9^^,^ 10 ^,^ 11 In this context, the aim of this animal study was to improve the comprehension of the mechanisms involved on the onset of CRS4 pathogenesis.

## MATERIAL AND METHODS

### ANIMAL MODEL

We used an animal model of renal dysfunction to analyze the myocardial damage in a CRS experiment. All experimental procedures were in strict accordance with our institutional guidelines and international standards for manipulation and care of laboratory animals, and were previously approved by the local Research Ethics Committee. We used Male Wistar rats, weighing about 250 g and the induction of CKD was performed under anesthesia with ketamine (Vetanarcol^R^ 50 mg/Kg, König) and xylazine (Anasedan^R^ 10 mg/Kg, Vetbands). A 5/6 nephrectomy was performed by removal of the right kidney and ligation of the appropriate left renal artery branches, thus ensuring the infarction of at least two-thirds of the left kidney to induce CKD as a one-step procedure. Sham-operated rats underwent anesthesia, ventral laparotomy, and manipulation of the renal pedicles, with no removal of renal mass. After recovering from anesthesia, the animals were returned to their original cages, given free access to tap water and standard chow (0.5% Na, 22% protein), and maintained at 23 ± 1°C under a 12:12-h light-dark cycle for a follow up period of 8 weeks. The animals were separated in SHAM group (n = 10) and CKD group (n = 31); the CKD animals were euthanized at 4 weeks (4W) and eight weeks (8W) after the surgery.

### BIOCHEMICAL ANALYSIS

Blood samples were collected by tail puncture after topic anesthesia at baseline, 4W, and 8W after the surgeries. The serum was obtained by centrifugation and the samples were stored in appropriate vials (*endotoxinfree*) at -20°C until analysis. Urea levels were determined in all samples through Endpoint Colorimetric Reaction Assay (Labtest^R^). Quantification of nt-proBNP and IL-6 serum levels were performed by the enzyme linked immunosorbent assay (ELISA - Elabscience Biotechnology BioLegend Inc. San Diego, CA) using optical density at 450 nm (ThermoplateMicroplate-TP reader). The TnI isoform was measured by chemiluminescent microparticles immunoassay (STAT hs troponin - Abbott Diagnostis).^12^

### HISTOLOGICAL ANALYSIS

Animals were euthanized and the hearts were removed and stored in formaldehyde. Histological sections were prepared to assess myocardial fibrosis and hypertrophy. Tissue *microarray* was performed to assess expression of TNF-α, nitrotyrosine, and angiotensin.

### STATISTICAL ANALYSIS

The results are reported as mean values ±SEM, with *p* < 0.05 indicating significance. The Tukey's multiple comparisons test and Friedman and Mann-Whitney U non-parametric tests were used to compare differences among groups. Also, the alpha was set at 0.05 and all tests were two-tailed. The IBM SPSS Statistics 20 software was used for analysis. The GhaphPad Prism 6 (GraphPad software, Inc, San Diego, CA) was used for the graphics.

## RESULTS

**Figure 1:**
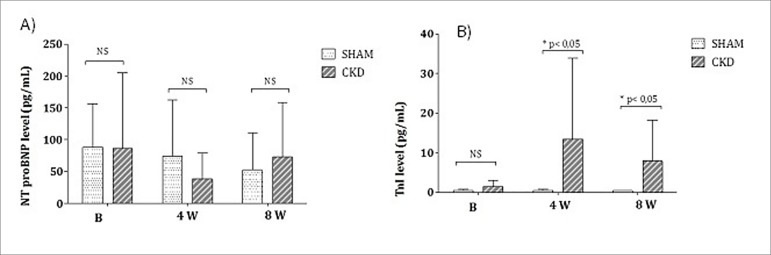
A) nt-proBNP levels and B) TnI levels.

There was no significant difference in animal body weight throughout the experiment between CKD and SHAM groups. Urea level was higher in the CKD group overtime compared to controls (*p* < 0.05). The analysis for nt-proBNP showed no significant difference between groups and between 4W and 8W. The comparisons of TnI levels showed a significant difference between the 4W and 8W (*p* < 0.05) when CKD was compared to SHAM (Figure 1).

**Figure 2:**
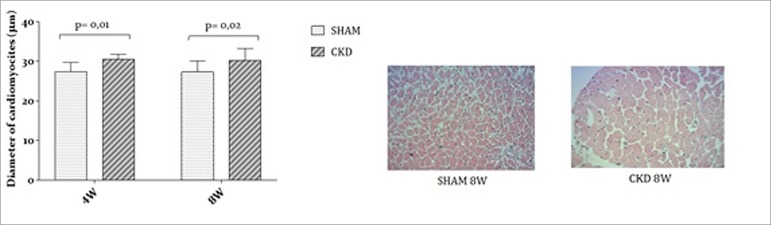
Comparison of myocardial hypertrophy between CKD and SHAM groups.

The heart weight was higher in the CKD group (1.79 ± 0.35 g) in comparison to SHAM group (1.46 ± 0.15 g) (*p* < 0.043). Figure 2 shows the myocardial hypertrophy of the CKD group with greater cardiomyocyte diameters than SHAM group (*p* < 0.001).

**Figure 3:**
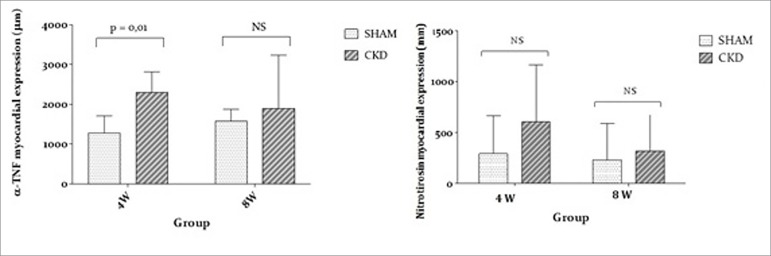
TNF-α and nitrotyrosine expressions for CKD and SHAM groups.

In relation to TNF-α expression, the highest levels occurred at 4W declining at the 8W evaluation. According to Figure 3, the increase in TNF-α in uremic animals was statistically significant at 4 weeks (*p* < 0.001). The statistical analysis of nitrotyrosine immunohistochemistry showed no significant difference between 4W and 8W.

**Figure 4:**
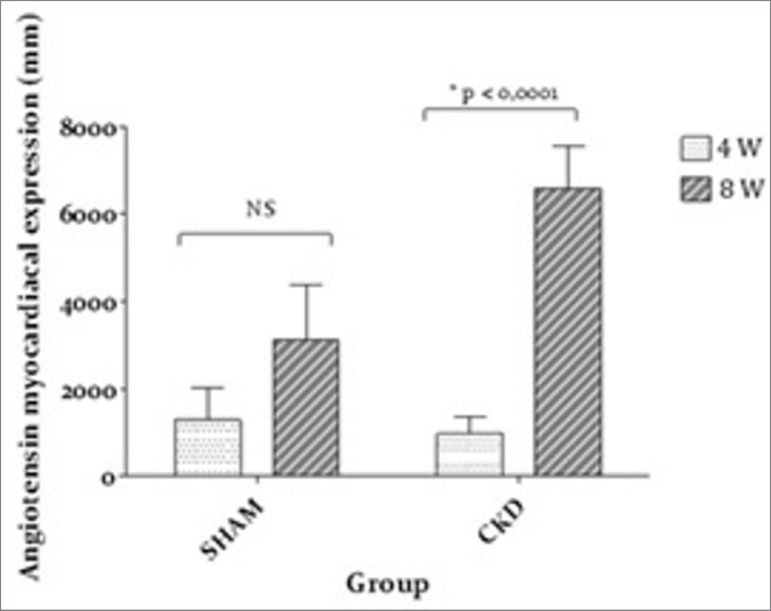
Angiotensin expression

As seen in Figure 4, a significant difference in angiotensin was found at 8W for the CKD group (*p* < 0.001).

**Figure 5:**
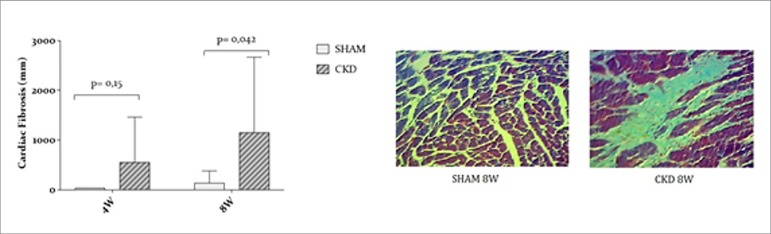
Myocardial fibrosis evaluations in CKD and SHAM groups.

Myocardial fibrosis presented no difference between groups at 4W (*p* = 0.15), however it was significantly more intense in the CKD group compared to SHAM at 8W (*p* = 0.042) suggesting that fibrosis increased over time (Figure 5).

## DISCUSSION

The 5/6 nephrectomy animal model causes an adaptive process with structural and functional hypertrophy of the remnant nephrons. It is considered a classic animal model for simulating a clinical situation.^13^^,^ 14 Systemic vascular resistance or expansion of the intravascular volume results in myocyte thickening and remodeling of the left ventricle. The CRS4 is probably independent of these factors, but they lead to the activation of apoptotic and autophagic cell signals that culminate in increased production of extracellular matrix and fibrosis. When hypertrophy reaches a threshold at which the increased muscle mass cannot compensate for the increased load, there is a hardening of the myocardial wall leading to ventricular fibrillation, fibrosis, hypertrophy, CRS4 development.^15^^-^ 17

The increased levels of cardiac biomarkers can be important predictors of mortality in CRS4.^18^ In our study, there was no difference in nt-proBNP levels at 4W and 8W comparing SHAM and CKD groups. The nt-proBNP is a marker of cardiac stretching and failure, provoked by a fluid overload found in cardiac insufficiency. Its elevation has been reported in various stages of CKD, with or without cardiovascular symptoms^19^^-^ 22 and levels increase as GFR decreases, being higher in the presence of cardiac failure.^17^ However, this alteration has not been developed or detected in the 5/6 nephrectomy animal model.

In CKD patients have higher levels of cardiac troponins when compared to non-CKD individuals and elevations are linked to worst prognosis.^23^ Few studies were published using troponins as markers of myocardial injury in CKD.^24^ Some authors have noted a high prevalence of increased levels of troponins in CKD in the absence of cardiovascular symptoms.^25^ There is no definitive established etiology for this increase; however, it seems to be a result of silent myocardial necrosis, left ventricular hypertrophy, endothelial dysfunction secondary to oxidative stress and inflammation, cardiac overload with distension, and others.^26^ In our study, there was an increase in TnI levels at 8W in CKD compared to SHAM group. This coincided with the decrease in kidney function, characterized by the expressive uremia. In a study by Fredericks *et al.* (2002), 8W after the 5/6 nephrectomy, rats showed significantly increased levels of TnI.^24^ Despite controversies, the persistently high levels of cardiac troponins in CKD individuals are not linked to impaired renal clearance, a widely known characteristic of this pathology, representing a myocardial injury biomarker. In addition, the troponin molecule is relatively big, which indicates that the kidney is not the main route for blood clearance.^23^ The improvement of renal function after replacement therapy does not change the high levels of cardiac troponin in CKD.^24^ Also, in a retrospective study assessing TnI levels after myocardial necrosis, the elimination and apparent half-life of TnI does not differ between individuals with normal kidney function and those in final stage CKD.^27^ We suggest that the increased levels of TnI at 8W in our study reflect the cardiac injury as a consequence of CKD progression in CRS4. The increased levels of TnI but not of nt-proBNP at 8W can be explained by their specific actions and cardiac alterations. TnI is linked to myocardial injury while nt-proBNP reflects stretching of cardiomyocytes with distinct mechanisms and causes.^3^^,^ 28 The adaptive changes that occur in the remaining nephrons resulting in hyperfiltration can influence nt-proBNP levels. The hemodynamic changes after the 5/6 nephrectomy are liked to structural glomerular lesions, that can be followed by proteinuria.^13^

The development of left ventricular hypertrophy (LVH) involves classic factors such as anemia, changes in renin-angiotensin-aldosterone system (RAAS), and hypertension in addition to the independent mechanisms from the mTOR, phosphorus, and parathyroid hormone (PTH). In our study, the heart weight increased and the results showed a hypertrophy when comparing CKD and SHAM groups. These data corroborate the literature about development of hypertrophy in CKD, independent of cardiac preload and post-load factors, but in the absence of hypertension or volume expansion by activation of cellular mTOR pathway. An animal model of CKD-related LVH found an activation of cellular mTOR pathway, even in the absence of pressure or volume expansion.^16^ Other experimental models and post-renal transplantation patients have shown that cell mTOR pathway was inhibited by the use of rapamycin (mTOR inhibitor partial), which led to a significant reduction in LV mass. Hyperparathyroidism and secondary hyperphosphatemia are being associated with LVH and probably involve similar pathways of mTOR activation.^29^^,^ 16 ^,^ 15

Several factors as inflammatory, oxidative stress, and injury can be involved in CRS4. The immune dysfunction in CKD patients leads to an accelerated tissue degeneration (as consequence of chronic inflammation) and increased rate of sepsis (because of a poor immune response) and are an important target to reduce mortality^6^ once inflammation is a cardio-renal connector for CRS4 development.^30^^,^ 31 The cytokine TNF-α is an important marker for inflammatory processes, being able to predict mortality linked to cardiovascular diseases in patients on dialysis.^32^ In our study, αTNF expression was increased in CKD, with a peak at 4W that was reduced at 8W. The same occurred with the TnI serum levels characterizing a connection between heart and kidneys that is present in CRS4. The development of a chronic inflammatory process is one of the key-points of connection between these two organs, as the injury of one can induce progressive impairment of the other, with imbalances between nitric oxide and reactive oxygen species.^30^ Our results did not show significant increase in nitrotyrosine but presented elevated plasma levels of lipid peroxidation and protein; reduction of antioxidant activity has been found in oxidative stress caused by the uremic state.^33^^,^ 34 According to other studies, several pathological conditions such as ischemia and inflammation can generate a high oxidant potential of peroxynitrite.^9^^-^ 11 ^,^ 35 We believe that other biomarkers may be used to assess the oxidative stress that are technically more sensitive than nitrotyrosine to evaluate CRS4.

The pathogenesis of CRS4 includes chronic activation of the RAAS and the sympathetic nervous system with reduced renal perfusion. Chronic activation of the RAAS can impair mitochondrial function and increase mitochondrial-derived oxidative stress, which in turn can lead to renal injury and sodium and water retention.^36^ In an experimental uremia and cardiac remodeling study, the isolated effects of hyperparathyroidism and phosphorus were found to be independently associated with major changes in cardiac remodeling process and LVE in CKD, and probably involve similar pathways to those related to mTOR activation.^37^ In our study, the angiotensin expression presented an increase, which corroborates the hypothesis that the development of an inflammatory process and increased activity of the renin-angiotensin axis can causes CRS4. Our study showed more intense myocardial fibrosis in 8W in CKD compared to SHAM and we suggest that fibrosis is increased overtime in the development of CRS4.

Therefore, this model showed that there is an inflammatory phenomenon that precedes the development of fibrosis in the natural history of CRS4. Despite the findings for nt-proBNP, the use of TnI can be a powerful tool for monitoring the cardiovascular and inflammatory consequences in CKD patients. Inflammation and activation of the RAAS system appear to be important phenomena in the induction of LVH and fibrosis that characterize CRS4. Concluding, this study reinforces the need for RAAS blockade as cardioprotective strategies and it emphasizes the need to control these factors in the CKD to avoid the development the CRS4.
